# Nutrition in Pregnancy and Early Life: A Critical Window of Vulnerability but Also of Opportunity

**DOI:** 10.3390/nu18050793

**Published:** 2026-02-28

**Authors:** Stefania Triunfo

**Affiliations:** Department of Obstetrics and Gynecology, ASST Santi Paolo Carlo, University of Milan, 20142 Milan, Italy; stefania.triunfo@unimi.it

According to the well-established fetal programming hypothesis proposed by Barker, consuming a balanced variety and an appropriate quantity of nutritious foods supports optimal fetal growth and improves both obstetric outcomes and infant health [1]. Beyond providing energy and substrates for tissue development, maternal nutrition modulates placental structure and function, metabolism, and inflammatory and endocrine pathways that collectively shape the intrauterine environment [2]. In this framework, pregnancy represents a critical window of vulnerability and opportunity. Therefore, exposures occurring during specific stages of gestation—such as early organogenesis or late fetal growth—may have disproportionate and long-lasting effects on offspring physiology [3].

Adverse environmental conditions during pregnancy, including undernutrition, excessive or imbalanced dietary intake, psychosocial stress, and exposure to toxicants, can elicit persistent biological adaptations in the fetus [4,5,6,7]. These adaptations are frequently mediated by epigenetic mechanisms (i.e., DNA methylation, histone modifications, and regulation by non-coding RNAs) that alter gene expression without changing the DNA sequence [8,9,10,11,12,13,14]. Predictive responses aimed at optimizing survival in the expected postnatal environment can be achieved by the fetus through these pathways. However, maladaptive adaptations may occur if the postnatal context differs significantly (such as nutrient scarcity in utero followed by postnatal rising intake), which can lead to an increase in cardiometabolic risk. This phenomenon, broadly termed developmental programming, has been associated with outcomes such as low birth weight, altered body composition, insulin resistance, hypertension, and neurobehavioral changes [1,2,3]. Importantly, evidence also suggests that some programming effects may extend beyond the directly exposed generation: when epigenetic marks escape erasure and re-establishment during gametogenesis and early embryonic development, altered regulatory states may be transmitted through the germline, contributing to intergenerational or transgenerational patterns of disease susceptibility [5]. For these reasons, pregnancy interventions aimed at optimizing the maternal environment are increasingly viewed as strategic tool, not only to reduce immediate obstetric complications, but also to mitigate longer-term consequences for offspring health and potentially limit transgenerational effects.

In fetal–maternal medicine and pediatrics, there has been a growing challenge for clinicians and researchers: substantial differences in dietary intake, eating patterns, and nutrition-related behaviors among pregnant populations [15,16,17,18,19,20,21]. This variability reflects socio-economic inequalities, such as food insecurity, limited access to high-quality foods, cultural and family norms, differing nutrition literacy, and broad changes in the contemporary food environment, including the widespread availability of energy-dense, nutrient-poor options. As a result, contrasting risk profiles may coexist within the same healthcare setting, ranging from inadequate micronutrient intake and insufficient gestational weight gain to excessive caloric intake, poor diet quality, and pregnancy complicated by overweight or obesity [6]. All these patterns can affect both maternal metabolic health and fetal growth trajectories by influencing both short-term outcomes and later-life risk for chronic disease.

Maternal nutrition interacts with a broader exposome: maternal and early-life exposure to environmental stressors and pollutants may compound biological vulnerability and influence endocrine, immune, and metabolic pathways relevant to pregnancy and fetal development [22,23,24]. Such exposures may also contribute to long-term alterations in reproductive and cardiometabolic health in offspring, further underscoring the complexity of maternal–infant wellbeing and the need for integrated preventive strategies. Overall, these considerations support a life-course approach that combines nutritional assessment, culturally tailored counseling, and attention to social and environmental determinants to improve health trajectories across generations.

This Special Issue, *Nutrition Strategy for Maternal and Infant Wellbeing*, aims to provide a multidisciplinary overview of nutritional determinants during pregnancy and lactation, and their associations with undernutrition, overweight and obesity, gestational hypertension, diabetes, metabolic syndrome, immune development, and long-term offspring health. The contributions published in this Special Issue collectively reinforce the importance of evidence-based nutritional strategies to optimize maternal and infant outcomes.

One important area addressed is maternal feeding practices and breastfeeding support. Gama and colleagues explored the breastfeeding experiences of Australian mothers of multiple birth infants, identifying unique barriers such as delayed initiation due to preterm birth, latching difficulties, perceived low milk supply, and the lack of tailored hospital guidance for mothers feeding twins or triplets [25]. Their findings emphasize the urgent need for specialized postpartum lactation support and structured guidelines for multiple birth families.

Breastfeeding intention and maternal support systems also remain critical determinants of breastfeeding duration. Anderson et al. demonstrated that prenatal breastfeeding intention is consistently associated with longer breastfeeding duration among women participating in the WIC program, highlighting the importance of strengthening prenatal education and family/community support mechanisms [26].

Several papers focused on the growing interest in microbiome-related nutritional interventions during pregnancy. Schwartz et al. investigated dietary fiber intake during the third trimester and its relationship with gut bacterial diversity. Their results suggest that most pregnant women fail to meet fiber recommendations, and that adequate intake may modestly improve microbiome diversity, underlining the need for nutritional improvement in pregnancy [27].

In parallel, Varlas and colleagues provided a systematic review of oral probiotics and their immune–metabolic influence during pregnancy and lactation. Reviewing over 15,000 women across randomized trials, the authors discussed the potential of probiotics to modulate glucose homeostasis, reduce excess gestational weight gain, and possibly prevent preeclampsia and allergic disease risk in offspring, although further research is needed to establish standardized recommendations [28].

The preventive role of probiotics in early-life allergic disease was also explored by Anania et al., who evaluated supplementation with *Bifidobacterium bifidum* PRL2010 during late pregnancy and breastfeeding. Although preliminary, their randomized trial suggests potential benefits in reducing the severity of atopic dermatitis manifestations in infants, supporting the growing interest in microbiota-targeted strategies [29].

Beyond microbiome interventions, micronutrient deficiencies remain a major concern [30,31]. Saluja et al. [32] examined vitamin D deficiency in a multi-ethnic cohort, demonstrating a strong association between low vitamin D levels and increased risk of gestational diabetes mellitus, particularly among South Asian women. These findings support the need for targeted screening and supplementation strategies in high-risk populations [32].

Chrono-nutrition has also emerged as a promising, physiologically grounded refinement of dietary management in gestational diabetes mellitus. Diurnal variations in insulin sensitivity and glucose tolerance may render meal timing and nutrient distribution clinically relevant. Summarizing observational and interventional evidence suggesting that later eating patterns and a higher evening carbohydrate load can worsen postprandial glycemic responses in women with diabetes. Although the literature remains heterogeneous and randomized controlled trials are still limited, this perspective highlights the potential of timing-based, low-risk dietary strategies as an adjunct to standard medical nutrition therapy [33].

Finally, the Special Issue also addresses culturally specific dietary practices and long-term reproductive health outcomes. Tran and colleagues investigated Ramadan fasting during pregnancy in Indonesia and found no association with offspring age at menarche, suggesting that mild intermittent fasting exposures may have limited impact on pubertal timing, although maternal nutrition remains a critical determinant of offspring health [34].

Taken together, the articles included in this Special Issue underscore the complexity of maternal nutrition, encompassing dietary quality, micronutrient adequacy, gut microbiome interactions, breastfeeding support, and culturally shaped nutritional behaviors. The evidence presented highlights the need for integrated nutritional policies and clinical strategies that support women throughout pregnancy and lactation. Moving forward, translating this growing body of evidence into personalized, equitable, and context-sensitive interventions will be essential to improve maternal wellbeing and promote healthier developmental trajectories for future generations.

Special acknowledgment must be given to all the authors for their valuable contributions, the reviewers for their constructive evaluations, and the *Nutrients* editorial team for their support in the development of this Special Issue.

The goal is for this collection to inspire further research and improve nutritional guidance and interventions for populations of childbearing age.
